# Possible impacts of the predominant *Bacillus* bacteria on the *Ophiocordyceps unilateralis s. l.* in its infected ant cadavers

**DOI:** 10.1038/s41598-021-02094-5

**Published:** 2021-11-22

**Authors:** Kai-Wen Tu, Ming-Chung Chiu, Wei-Jiun Lin, Yen-Ping Hsueh, Chung-Chi Lin, Jui-Yu Chou

**Affiliations:** 1 Department of Biology, National Changhua University of Education, Changhua 500, Taiwan; 2 Institute of Molecular Biology, Academia Sinica, Taipei, Taiwan

**Keywords:** Ecology, Microbial ecology

## Abstract

Animal hosts infected and killed by parasitoid fungi become nutrient-rich cadavers for saprophytes. Bacteria adapted to colonization of parasitoid fungi can be selected and can predominate in the cadavers, actions that consequently impact the fitness of the parasitoid fungi. In Taiwan, the zombie fungus, *Ophiocordyceps unilateralis *sensu lato (Clavicipitaceae: Hypocreales), was found to parasitize eight ant species, with preference for a principal host, *Polyrhachis moesta*. In this study, ant cadavers grew a fungal stroma that was predominated by *Bacillus cereus/thuringiensis*. The bacterial diversity in the principal ant host was found to be lower than the bacterial diversity in alternative hosts, a situation that might enhance the impact of *B. cereus/thuringiensis* on the sympatric fungus. The *B. cereus/thuringiensis* isolates from fungal stroma displayed higher resistance to a specific naphthoquinone (plumbagin) than sympatric bacteria from the environment. Naphthoquinones are known to be produced by *O. unilateralis s. l.*, and hence the resistance displayed by *B. cereus/thuringiensis* isolates to these compounds suggests an advantage to *B. cereus/thuringiensis* to grow in the ant cadaver. Bacteria proliferating in the ant cadaver inevitably compete for resources with the fungus. However, the *B. cereus/thuringiensis* isolates displayed in vitro capabilities of hemolysis, production of hydrolytic enzymes, and antagonistic effects to co-cultured nematodes and entomopathogenic fungi. Thus, co-infection with *B. cereus/thuringiensis* offers potential benefits to the zombie fungus in killing the host under favorable conditions for reproduction, digesting the host tissue, and protecting the cadaver from being taken over by other consumers. With these potential benefits, the synergistic effect of *B. cereus/thuringiensis* on *O. unilateralis* infection is noteworthy given the competitive relationship of these two organisms sharing the same resource.

## Introduction

Fungi and bacteria often live in close proximity and share the same microhabitats. The inevitable competition for limited resources promotes the selection of partners tolerant to the presence of each other. Ecological interactions that are either antagonistic or synergistic can develop. Competition for the same resources enhances the antagonistic relationship in many fungus–bacterium associations^1^. However, fungi and bacteria can co-occur synergistically, enhancing their partner's adaptations and consequently forming a co-evolving metaorganism^2^.

The ant-pathogenic fungus, *Ophiocordyceps unilateralis *sensu lato, is a well-known parasitoid that causes manipulated behaviors and subsequent death of the ant hosts^3^. Spores invade the ant hosts by attaching, germinating, and penetrating the cuticles of foraging ant workers. The fungus lives in a yeast-like state as single cells in the host hemolymph and causes a series of host behavioral manipulations, which is the origin of the name “zombie ant.” These include convulsion, erratic walking, and finally, the host dying after biting onto leaf veins or twigs. The fungus produces hyphae to form a branching network of cells throughout the ant cadaver^4^, exploits resources by lysing the host tissue, and ends the parasitic life cycle with a stroma sprouting from the intersegmental membrane between the head and prothorax of the host, forming the perithecial plates for spreading spores^5^.

During the ~ 1–2 weeks in which hyphal development occurs in the ant cadaver^5^, the fungus is under selective pressure for host resource exploitation and defense against invaders. Animal cadavers in the terrestrial environment are a nutrient-rich resource for saprophytes^6^. The scarcity of cadavers due to patchy distribution, transient nature, and short supply^7^ promotes rapid colonization and consumption of the available resource by bacteria, which results in a quick succession of microbial populations^8^. However, fungal colonization renders the resource unavailable for most bacteria. Fungal products, such as penicillin^9^, usually result in significantly reduced microbial biodiversity. In the case of *O. unilateralis s. l.*-infected ant cadavers, naphthoquinone derivatives are produced by *O. unilateralis* and affect growth of sympatric bacteria negatively^10–12^. Bacterial communities shaped by fungal colonization have been characterized in various environments and the specific impacts on both organisms have been described^2^. However, bacterial communities in insect cadavers colonized by parasitoid fungi have been described less frequently^13,14^.

In the present study, we describe the bacterial communities in *O. unilateralis s. l.*-infected ant cadavers. Because the host range is wider in *O. unilateralis s. l.* from Taiwan^15^ compared with related species^16–19^, bacterial communities were captured by a culture-dependent method from two infected ant species, a principal host, *Polyrhachis moesta*, and an alternate sympatric host, *P. wolfi*. We evaluate the biological properties and the possible impact of the predominant bacterial species on *O. unilateralis s. l.* infection and examine bacterial growth in relation to the production of naphthoquinone derivatives by *O. unilateralis s. l.*^10–12^*.* We further investigate the impact on *O. unilateralis s. l.* infection by examining the bacterial species for biological properties related to host-killing (hemolysis and presence of pathogenic and antibiotic genes), resource exploitation (capabilities for lysing host tissue), and defense against possible invaders of the host cadaver (lethal effects on scavenger nematodes and antagonism toward entomopathogenic fungi).

## Materials and methods

### Sample collection

**Figure 1:**
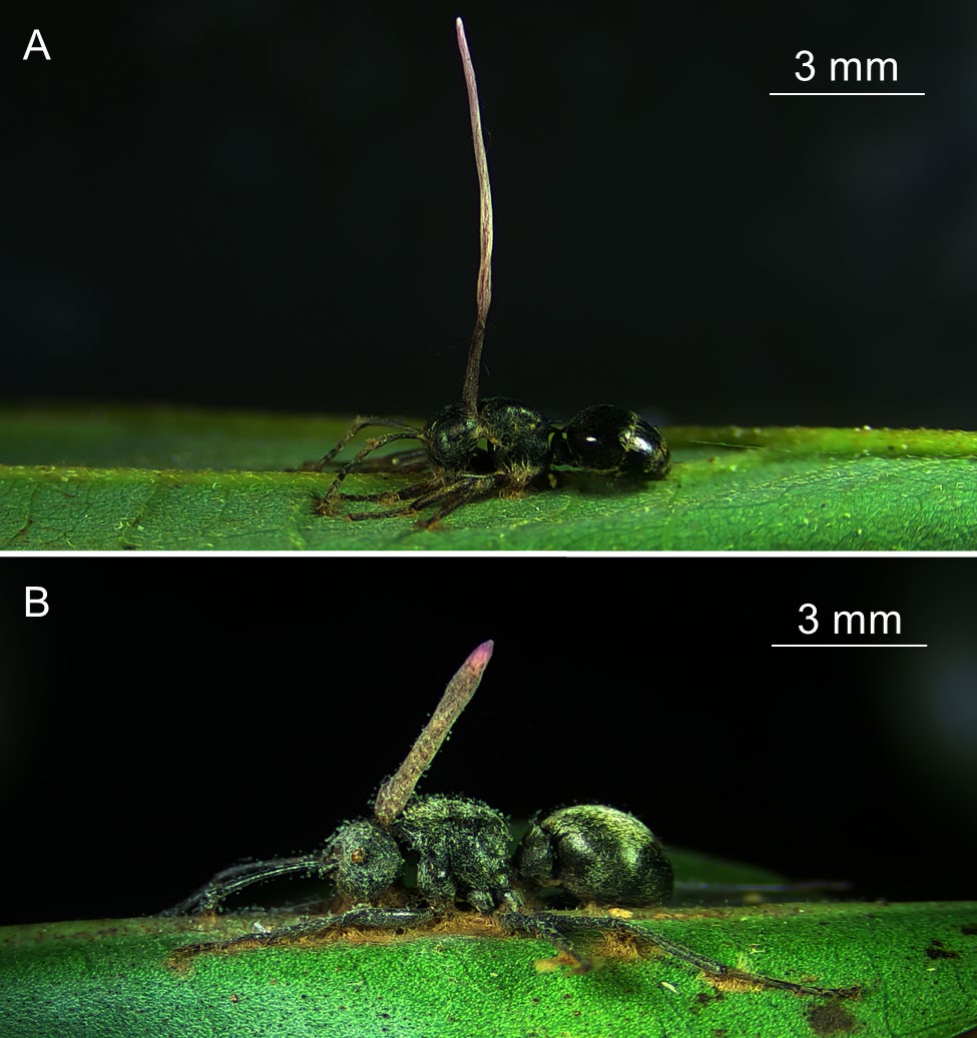
*Ophiocordyceps unilateralis *sensu lato-infected (**a**) *Polyrhachis moesta* and (**b**) *P*. *wolfi*, with the stroma growing from the ant cadaver. The specimens were collected from the Lianhuachi Research Center, Taiwan and photographed in the laboratory by Wei-Jiun Lin.

Samples were collected from an evergreen broadleaf forest in central Taiwan (Lianhuachi Experimental Forest, Nantou County, 23°55′7″N 120°52′58″E) from January 2017 to March 2018. Permission to collect plants for the study was obtained from the Lianhuachi Research Center, Taiwan Forestry Research Institute, Council of Agriculture, Executive Yuan, Taiwan (Permission no.: 1062272538). The present study complies with the International Union for Conservation of Nature Policy Statement on Research Involving Species at Risk of Extinction and the Convention on the Trade in Endangered Species of Wild Fauna and Flora. Ant cadavers with fungal growth were collected from understory plants with a height of less than 3 m. Ant cadavers infected with *O*. *unilateralis s*. *l*. were removed carefully by cutting the leaf and placing it into a 50-mL conical centrifuge tube, which was then transported to the laboratory. Only cadavers in which the fungal growth stage preceded the development of perithecia, which theoretically has the highest biological activity, were collected (Fig. 1). In total, 24 infected *P. moesta* and 20 infected *P. wolfi* samples were collected.

### Isolation and cultivation of bacteria

Ants on the leaves were first identified to species and then, using tweezers, each ant was placed carefully into a sterilized 1.5-mL microcentrifuge tube [see details in Lin et al. (2020)^15^. Samples were shaken one by one in 600 μL of sterilized water for a few seconds at 3000 revolutions/min (rpm) using a vortex mixer (AL-VTX3000L, CAE technology Co., Ltd., Québec, Canada), and were then soaked with 600 μL of 70% ethanol to sterilize the ant’s surface. The ethanol on the samples was washed twice with 600 μL of sterilized water, then vortexed in 400 μL of sterilized water. Next, 200 μL of the supernatant was spread homogeneously onto a Luria–Bertani (LB) agar plate (25 g Luria–Bertani broth and 15 g agar per liter) to confirm the absence of live bacteria.

Bacteria from inside the ant host were released by homogenizing the ant host in 200 μL of water and culturing on LB agar plates at 28 °C for 2 days. Bacteria from each of the ant individuals were cultured independently and approximately equal numbers of the isolates were picked randomly with sterile toothpicks, and were suspended in the LB medium supplemented with 15% v/v glycerol and maintained at − 80 °C until the time of examination. In total, 247 bacterial isolates from *P. moesta* and 241 bacterial isolates from *P. wolfi* were collected.

In addition to the bacterial isolates from the ant bodies, 60 bacterial isolates from soil, leaves, and air in the same forest were collected for the purpose of comparing their resistance to naphthoquinones (see below) by using the aforementioned procedure but without initial cleaning and sterilizing of the sample surface.

### Bacterial identification

Bacteria collected from the ant hosts were identified by gene marker sequencing. Bacterial isolates were cultured in LB medium at 28 °C overnight to reach the log-phase, and genomic DNA was extracted following the methods described in Vingataramin and Frost (2015)^20^. Conspecies/strains of the bacterial isolates from the same host were determined using the randomly amplified polymorphic DNA (RAPD) method with the primer 5′-GAGGGTGGCGGTTCT-3′. PCR amplification was performed as follows: initial denaturation at 95 °C for 5 min, 40 cycles of amplification including denaturation at 95 °C for 1 min, annealing at 42 °C for 30 s, and extension at 72 °C for 1 min, followed by a final extension at 72 °C for 10 min. PCR products were run in 2% agarose gel and bacterial isolates were characterized by fragment patterns. For each of the ant hosts, bacterial isolates with the same RAPD pattern were considered to be the same strain. In total, 106 and 178 strains were found from *P. moesta and P. wolfi*, respectively. One of the bacterial isolates was selected at random to represent the strain and coded with “JYCB” followed by a series of numbers (e.g., JYCB191). Taxonomic status of each strain was determined to species by using the V3/V4 region of the 16S rDNA gene. PCR amplification with the primer set (8F: 5′-AGAGTTTGATCCTGGCTCAG-3′ and 1541R: 5′-AAGGAGGTGATCCAGCCGCA-3′)^21,22^ was performed under the following conditions: initial denaturation at 95 °C for 5 min, 40 cycles of amplification including denaturation at 95 °C for 1 min, annealing at 55 °C for 30 s, and extension at 72 °C for 1 min 45 s, followed by a final extension at 72 °C for 10 min. PCR products were first checked by running a gel, and were then sequenced at Genomics, Inc. (New Taipei City, Taiwan).

The sequences of the bacterial strains from each of the ant hosts were first analyzed by the unweighted pair group method with arithmetic mean (UPGMA) analysis and clustered into clades according to the sequence dissimilarity (< 0.01) by using MEGA X^23^. Species of each of the clades were judged by the basic local alignment search tool (BLAST) method against nucleotide sequences in the National Center for Biotechnology Information (NCBI) nucleotide database (https://ftp.ncbi.nlm.nih.gov/blast/db/), updated through 2021 May 17th. Because each of the clades contains one to several bacterial strains, each of the strains was first labeled by the species of the sequence with the highest BLAST identity, which was ranked by expected value, percentage of identical matches, and alignment length (https://www.ncbi.nlm.nih.gov/BLAST/tutorial/Altschul-1.html). If multiple sequences from the database were found to be same in the indexes of the identity, the bacterial strain was labeled by the species that appeared most frequently. Finally, the species for each of the clades were judged by the bacterial species found most frequently in the strains belonging to the clade.

The 60 bacterial isolates collected from the environment were examined using the RAPD method and a *Bacillus*-specific primer set (5′-CTTGCTCCTCTGAAGT TAGCGGCG-3′ and 5′-TGTTCTTCCCTAATAACAGAGTTTTACGACCCG-3′), with PCR conditions suggested in Nakano et al. (2004)^24^. Twenty of the bacterial isolates (10 *Bacillus* and 10 non-*Bacillus*) with different RAPD patterns were collected for further experiments.

### Bacterial diversity of the two ant host species

Three biodiversity indexes (Chao1 richness, exponential of Shannon entropy, and inverse Simpson concentration) of bacterial species were estimated by the sample size-based rarefaction/extrapolation sampling curve using the abundance of bacterial isolates from each of the two ant host species^25^. The calculation was conducted using R^26^ with the “iNEXT” package^27^.

### Biological properties of bacterial isolates from infected ants

#### Selected strains

For examining the biological properties of the most predominant species, *B. cereus/thuringiensis* (see results), 11 of 47 *B. cereus/thuringiensis* strains from *P. moesta* and 10 of 63 *B. cereus/thuringiensis* strains from *P. wolfi* were selected. The strains were selected according to the UPGMA analysis of the sequence. One to three strains grouped in the same cluster were selected (Fig. S1). In addition, 6 of 15 strains of the second-most predominant *Bacillus* species (*B. gibsonii*) in *P. wolfi* were also selected randomly for examination, because *B. gibsonii* occupied approximately 20% of the individuals within the *Bacillus* isolates.

All the selected strains were used to examine biological properties including potential (1) capability of the isolate to lyse host tissue (hydrolytic enzymes); (2) defense against fungal competition for the ant cadaver, involving the presence of pathogenic and antibiotic genes; and (3) resistance to naphthoquinone derivatives. In addition to the repellence against entomopathogenic fungi, one strain of the *B. cereus/thuringiensis* and one strain from the secondarily predominant *Bacillus* clade from each of the hosts were selected at random for examining the potential impact on the invasion and consumption of ant cadavers by scavenger nematodes.

#### Hemolysis reaction

Hemolysis reaction tests were conducted on tryptic soy agar (TSA) plates (15 g pancreatic digest of casein, 5 g soybean meal, 5 g NaCl, and 15 g agar, with final pH of 7.3) mixed with 5% defibrinated sheep blood, which was added to the TSA after it had cooled down to approximately 50 °C. One 3-µL drop of the log-phase bacterial suspension was placed onto each TSA plate and incubated at 28 °C for 1–2 days.

The hemolysis reaction was determined by the formation of clean (β-hemolysis) or greenish (α-hemolysis) hemolytic zones, or no such zone (γ-hemolysis, non-hemolytic) around the bacterial colonies^28^.

#### Production of hydrolytic enzymes

The production of hydrolytic enzymes was examined by culturing a 3-µL drop of the exponential-phase bacterial suspension on four different types of plated media: chitinase detection medium (solid medium with 0.3 g MgSO_4_.7H_2_O, 3 g (NH_4_)_2_SO_4_, 2 g KH_2_PO_4_, 1 g citric acid monohydrate, 0.15 g bromocresol purple, 200 μL Tween 80, 4.5 g colloidal chitin, and 1 L deionized water with 1.5% [w/v] agar, with final pH of 4.7); skim milk agar (solid medium with 2% [w/v] agar, 28 g skim milk powder, 5 g casein enzymic hydrolysate (Tryptone), 2.5 g yeast extract, 1 g dextrose, and 1 L deionized water); lipase agar (solid medium with 2% [w/v] agar, 0.1 g phenol red, 1 g CaCl_2_, 10 mL olive oil, and 1 L deionized water, with final pH of 7.4); and esterase agar (solid medium with 2% [w/v] agar, 0.1 g phenol red, 1 g CaCl_2_, 10 mL tributyrin, and 1 L deionized water, with final pH of 7.4). The chitinase detection medium was used to examine purple zones, indicating chitinase activity^29,30^; the skim milk agar medium was used to examine clearance zones for proteases activity^31^; and the lipase and esterase agar media were used to examine yellow zones, indicating lipase and esterase activity, respectively^32^.

#### Pathogenic and antibiotic genes

The total genomic DNA of *Bacillus* strains was extracted by using an AccuPrep genomic DNA extraction kit (Bioneer, Daejeon, Korea) for PCR amplification. The specific screening primers for amplifying the genes, including *cry*, *cyt*, Iturin, Chitinase, Bacillomycin, Fengycin, Surfactin, *vip*, and Zwittermicin A, were used under PCR conditions suggested in previous studies^33–36^. The primer sets used for the amplifications are listed in Table S2.

#### Lethal effects on Caenorhabditis elegans

Antagonistic effects of *B. cereus/thuringiensis* isolates on the model nematode, *C. elegans*, were examined by estimating the potential of hemolytic *B. cereus/thuringiensis* to prevent competition by scavengers for the resource-rich insect cadavers^37^. Daily mortality of *C*. *elegans* strain N2 was compared between randomly selected *B. cereus/thuringiensis* strains (*B. cereus/thuringiensis* JYCB227 in clade m4 from *P. moesta* and *B. cereus/thuringiensis* JYCB302 in clade w4 from *P. wolfi*) and *Bacillus* species of secondary predominance (*Bacillus* sp. JYCB252 in clade m8 from *P. moesta* and *B. gibsonii* JYCB395 in clade w30 from *P. wolfi*).

Synchronized fourth-stage larval (L4) nematodes were grown on nematode growth medium (NGM) (3 g NaCl, 2.5 g peptone, 17 g agar, 5 mg cholesterol, 1 mL 1 M CaCl_2_, 1 mL 1 M MgSO_4_, 25 mL 1 M KH_2_PO_4_, and H_2_O to 1 L) agar plates seeded with *Escherichia coli* OP50. The *Bacillus* isolates were prepared by inoculating in 3 mL LB liquid broth at 20 °C (the mean annual temperature in Lianhuachi Research Center, where the infected ants were collected) overnight, and then adjusting to an absorbance of optical density (O.D.) 0.2 at a wavelength of 600 nm.

To test the survival rate of *C. elegans* in the presence of various bacteria, L4 nematodes were co-cultured with (1) a hemolytic bacterial strain; (2) a non-hemolytic bacterial strain; (3) a hemolytic strain + *E. coli* OP50; (4) a non-hemolytic strain + *E*. *coli* OP50; and (5) *E*. *coli* OP50 only (control). We added 20 μL of bacterial culture to a 35-mm NGM agar plate and spread evenly with a glass rod. For each treatment, 30 L4 larvae were cultured on the NGM agar plate and their survival was monitored daily for 7 days. Each treatment was replicated three times.

Survival curves were compared using a survival analysis with treatment as the fixed effect. The significance of fixed effect was assessed by model reduction and the likelihood ratio test. Post-hoc multiple comparisons were conducted with Tukey’s all-pair comparisons. The model building and hypothesis tests were conducted by using the“survival” and “multcomp” packages in R.

#### Antagonism to entomopathogenic fungi

We examined the response of three entomopathogenic fungi, including *Aspergillus nomius* (isolated from the ant *Dolichoderus thoracicus*), *Trichoderma asperellum* (isolated from the litchi stink bug, *Tessaratoma papillosa*), and *Purpureocillium lilacinum* (isolated from *T. papillosa*), to co-cultured *Bacillus* strains. The entomopathogenic fungi were prepared by culturing on potato dextrose agar plates for 4 (*A. nomius*, *T. asperellum*) or 10 (*P. lilacinum*) days at 28 °C, until the mycelia covered approximately 80% of the plate.

A piece of mycelium (approximately 5 × 5 mm^2^) was seeded in the center of a TSA plate and surrounded by three equidistant 3-μL drops of exponential-phase bacterial suspension. Plates were incubated at 20 °C for 7–10 days. After incubation, areas of the mycelium occupying the plate surface were photographed and measured using Image J. Each pair of bacteria and entomopathogenic fungi, plus the control (a piece of mycelium not surrounded by the bacterial suspension) was replicated 3–4 times.

Antagonism was estimated based on the percentage of mycelial growth inhibition (MGI), which was calculated using the formula ([R_c_ − R_exp_]/R_c_) × 100%, where R_c_ is the mean area of the control fungus and R_exp_ is the mean area of the examined entomopathogenic fungus co-cultured with each of the *Bacillus* strains^38^. The MGI value from each of the bacteria co-cultured with each of the fungi was first tested by using Student’s *t* test and Holm–Bonferroni method to adjust *P* values. The MGI values among all entomopathogenic fungi were compared using a beta regression model with the *Bacillus* species as the fixed effect. The significance of the *Bacillus* species effect was tested by comparing the full model with a model that removed the fixed effect term by using a likelihood ratio test. Post-hoc tests were conducted using a Tukey-adjusted pairwise comparison. The statistical analysis was conducted using the R packages “betareg,” “emmeans,” “lmtest,” and “multcomp.”

#### Resistance of bacterial isolates to naphthoquinones

To examine the resistance of bacterial isolates to naphthoquinones, the growth of 11 predominant *B. cereus/thuringiensis* strains isolated from the principal ant host was compared with the growth of 20 environmental bacterial isolates (10 *Bacillus* and 10 non-*Bacillus*) using two naphthoquinones, respectively. Because fungal naphthoquinones are currently not purified and commercialized, the two naphthoquinones prepared for the experiment, plumbagin^39^ and lapachol^40^, were those found in plants. They were dissolved in a 30% dimethyl sulfoxide (DMSO) water solution^39^. Naphthoquinone concentrations were determined from the serial dilutions in which three randomly selected bacteria from the ant host and three from the environment had the most distinctive growth rate.

In this experiment, the bacterial isolates were first inoculated in LB medium at 20 °C overnight and were then refreshed to the exponential phase with LB medium for 3 h. The bacterial concentration was adjusted to ~ 1.5 × 10^8^ cells/mL. Next, 10 μL of the bacterial suspension and 180 μL of the Mueller Hinton broth medium (Sigma-Aldrich, St. Louis, USA) were added to either 10 μL of the naphthoquinone solution or 10 μL of the 30% DMSO water solution for the control. The growth of bacterial isolates at 20 °C was monitored by measuring the O.D. value at 600 nm with a Multiskan GO microplate spectrophotometer (Thermo Scientific, Waltham, USA) every hour for 12 h. Four bacterial isolates (one *B. cereus/thuringiensis* from the ant host, plus two *Bacillus* and one non-*Bacillus* from the environment) were omitted from the analysis due to low growth rate in the media with DMSO (O.D. value lower than 0.05 at the end of 12 h). Thus, ten predominant *Bacillus* from the ant, eight *Bacillus* from the environment*,* and nine non-*Bacillus* bacteria from the environment were used to represent the naphthoquinone tolerances of each group. Each combination of bacterial isolate and naphthoquinone or control was replicated twice.

The resistance index of each bacterial isolate was calculated by the normalized difference of the O.D. value in the naphthoquinone-treated medium versus the control medium ([naphthoquinone − DMSO]/[naphthoquinone + DMSO]). Values closer to 1 represent higher resistance to the presence of naphthoquinone. Resistance index was compared among the bacterial isolates from different resources (*B. cereus/thuringiensis* from the ant host, *Bacillus* from the environment, and non-*Bacillus* from the environment) using a linear mixed model with resource as the fixed effect, bacterial isolate as a random effect, and growth time (5–12 h) as a nest effect. The significance of resource as a fixed effect was assessed by model reduction and the likelihood ratio test. Post-hoc multiple comparisons were made using Tukey’s all-pair comparisons. The model building and hypothesis tests were conducted using the “lme4” and “multcomp” packages in R.

## Results

### Relative abundance and diversity of cultivated bacteria in infected ant hosts

In total, 247 and 241 bacterial isolates were obtained, with 106 and 178 strains identified by the RAPD patterns, from infected *P. moesta* and *P. wolfi*, respectively. The 16S rDNA partial sequences for each of the strains were uploaded to the NCBI database with the Genbank accession numbers provided in Supplementary file 1 and 2. According to the UPGMA results, the bacterial strains from the two hosts were clustered into 31 and 37 clades, respectively (Fig. S2).

**Figure 2:**
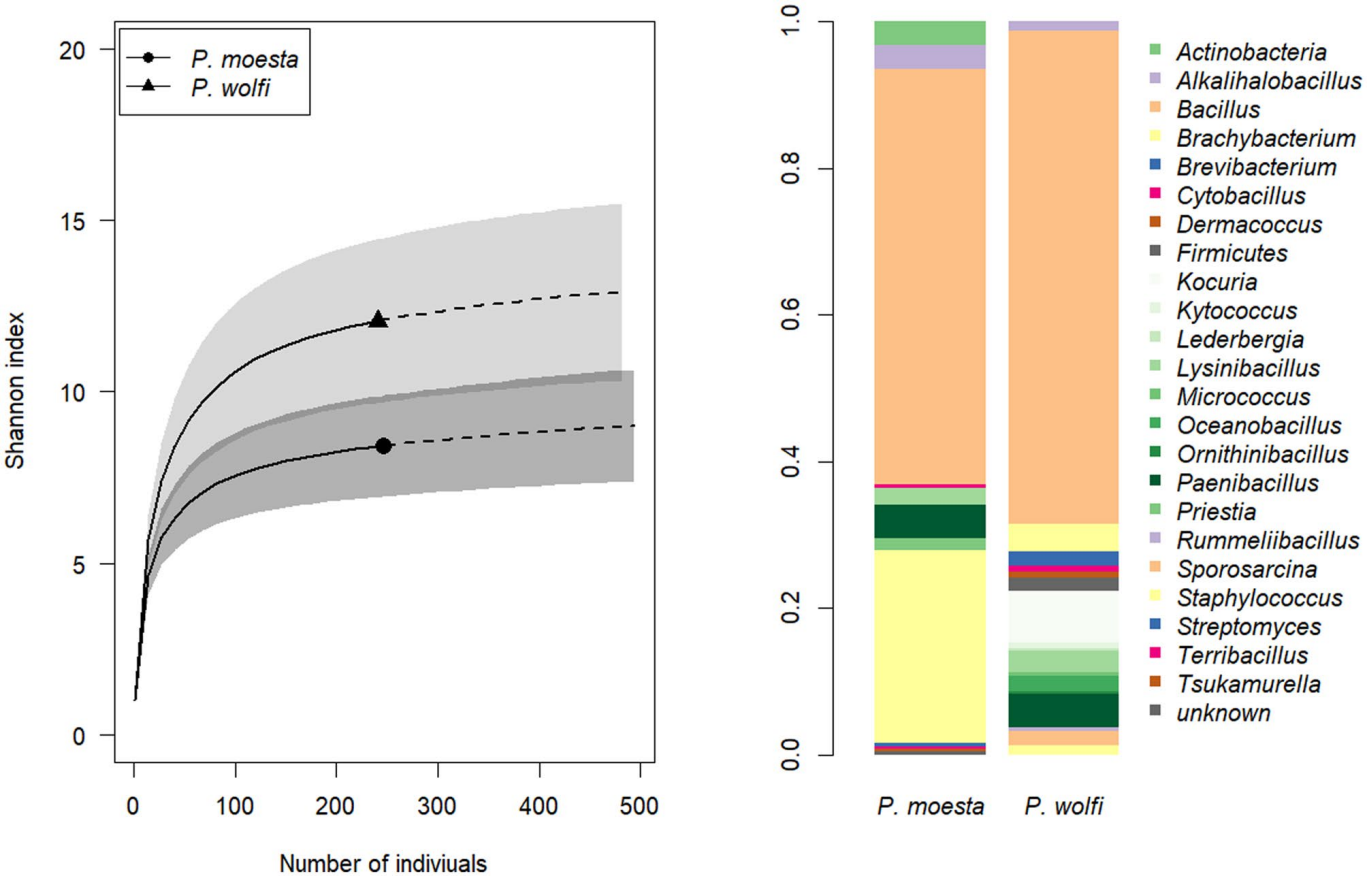
Species diversity and genus abundance of bacteria isolated from ant cadavers infected with *Ophiocordyceps unilateralis *sensu lato estimated by using R software with the “iNEXT” package (https://www.r-project.org/).

All the bacteria identified from the ant hosts belonged to the phyla Firmicutes and Actinobacteria. Ten genera were identified in *P. moesta* and 17 genera were identified in *P. wolfi*, while six genera were found commonly in both hosts (Table S1, Fig. 2). *Bacillus* was the most predominant genus in both species of ants. *Bacillus* comprised 56.68% (140/247) of total bacterial isolates from *P. moesta* and 65.98% (159/241) of total bacterial isolates from *P. wolfi*. We note that the bacteria identified as *Bacillus* might not be a monophyletic group based on UPGMA results. Five (m7, m8, m11, m13, m18) of the nine “*Bacillus*” clades from *P. moesta*, and four (w11, w12, w20, w30) of the eight from *P. wolfi* were not clustered into the same clade with other *Bacillus*. Despite this, excluding these clades from *Bacillus* did not change the predominance of *Bacillus* in the bacterial community because most of the *Bacillus* clades (except m4, w4, w8, w30) were low in abundance, with less than 10 individual isolates. Two *Bacillus* clades (m4 and w4) from each of the ant hosts were the most abundant, comprising 44.94% and 39.00% of the total bacterial isolates in *P. moesta* and *P. wolfi*, respectively, whereas w8 (*B. subtilis*) and w30 (*B. gibsonii*) comprised 6.22% and 14.11% isolates. The species of the predominant clades (m4 and w4) was considered to be *B. cereus/thuringiensis*. Most of the bacterial strains belonging to these two clades were labeled as *B. thuringiensis* (22/47 in m4, 42/69 in w4) according to the BLAST results. However, these clades also contained some strains labeled as *B. cereus* (16/47 in m4, 17/69 in w4). Although most of the strains are suggested to be *B. thuringiensis*, the 16S rRNA gene sequences based on universal primers have shown a high similarity (> 99%) index between *B. cereus* and *B. thuringiensis*^41^*.* It is difficult to differentiate *B*. *cereus* from *B. thuringiensis* in routine diagnostics. The identification methods are expensive and laborious because current species designation is linked to specific phenotypic characteristics or the presence of species-specific genes^42^. Because the identification was done according to the 16S rRNA gene sequence only in this study, these clades were considered as *B. cereus/thuringiensis* rather than one or the other.

The number of the bacterial clades in infected *P. wolfi* (37 species, estimated sample coverage: 94.63%) was more than that in infected *P. moesta* (31 species, estimated sample coverage: 93.93%). Sample size-based rarefaction and extrapolation curves also showed a higher diversity of microbiota in infected *P. wolfi* in all three biodiversity indexes, whereas the difference in species richness was not as obvious compared with the other two indexes (Figs. 2, S4).

### Hemolytic activity of bacterial isolates

In the 106 bacterial strains from *P. moesta,* 51 (48.11%) displayed the β-hemolysis reaction, whereas in the 178 bacterial strains from *P. wolfi*, 73 (41.01%) displayed the β-hemolysis reaction. None of the isolates displayed an α-hemolysis reaction. Among the bacteria belonging to the phylum Actinobacteria, none are hemolytic. Most of the strains belonging to the predominant clade, *B. cereus/thuringiensis*, displayed the β-hemolysis reaction, whereas 44 out of the 47 isolates from *P. moesta* and 63 out of the 69 isolates from *P. wolfi* were hemolytic. Details of the hemolysis of the bacterial isolates are listed in Table S1 and Supplementary file 1 and 2.

### Production of hydrolytic enzymes by *B. cereus/thuringiensis* and *B. gibsonii*

*B. cereus/thuringiensis* isolates from both ant hosts displayed protease, lipase, and esterase activities. Chitinase activity was detected in *B. cereus/thuringiensis* isolates from *P. wolfi*, but was not confirmed in the *B. cereus/thuringiensis* isolates from *P. moesta* because none of these isolates grew on the chitinase detection medium. Lipase activity was detected in all of the *B. gibsonii* isolates, but none of the isolates grew on either the chitinase detection medium, skim milk agar (for protease activity), or esterase agar plates (Table S5).

### PCR-based screening of endotoxin-related and biosynthetic genes

Most of the pathogenic and antibiotic genes examined in the current study were not detected commonly in the 11 *B. cereus/thuringiensis* strains from *P. moesta*, 10 *B. cereus/thuringiensis* strains from *P. wolfi*, or 6 *B. gibsonii* strains from *P. wolfi*. In the examination of *cry* genes, one *B. gibsonii* strain displayed the signal for the *cry2* gene, and two *B. cereus/thuringiensis* strains from *P. moesta* displayed the signal for the *cry3* gene. The chitinase gene signal was displayed in four *B. cereus/thuringiensis* strains from *P. moesta*, two *B. cereus/thuringiensis* strains from *P. wolfi*, and one *B. gibsonii* strain. The Surfactin gene signal was displayed in four *B. cereus/thuringiensis* strains from *P. moesta*. The Zwittermicin A gene signal was displayed in one *B. cereus/thuringiensis* strain from *P. moesta*. The other five genes examined (*cyt*, Iturin, Bacillomycin, Fengycin, and *vip*) were not detected in any of the *Bacillus* strains (Tables S3, S4).

### Potential impact of *B. cereus/thuringiensis* on *C. elegans* fitness

**Figure 3:**
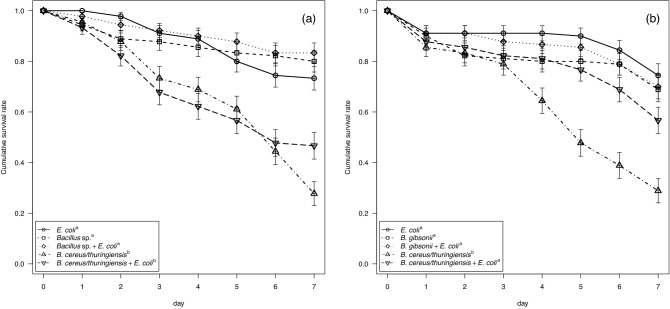
Daily cumulative survival rate (% ± SE) of the nematode, *Caenorhabditis elegans*, co-cultured with hemolytic and non-hemolytic bacteria isolated from ant cadavers of (**a**) the principal host, *Polyrhachis moesta*, and (**b**) the alternative host, *P. wolfi*, infected with *Ophiocordyceps unilateralis *sensu lato. *Bacillus* strains used in the examination were JYCB227 in clade m4 (*B. cereus/thuringiensis*) and JYCB252 in clade m8 (*Bacillus* sp.) from *P. moesta*, and JYCB302 in clade w4 (*B. cereus/thuringiensis*) and JYCB395 in clade w30 (*B. gibsonii*) from *P. wolfi*. Letters indicate significant pairwise differences among the treatments (Tukey’s all-pair comparisons using the beta regression model, *p* < 0.05).

Hemolytic *B. cereus/thuringiensis* from both ant hosts displayed higher mortality to nematodes than did the coexisting non-hemolytic *Bacillus*, whereas non-hemolytic *Bacillus* displayed mortality to nematodes at a level similar to that of the *E. coli* OP50 control (*P. moesta*: *X*^*2*^ = 80.14, *d.f.* = 4, *P* < 0.001, Fig. 3a; *P. wolfi*: *X*^*2*^ = 46.39, *d.f.* = 4, *P* < 0.001, Fig. 3b). The addition of *E. coli* OP50 slightly increased the survival rate of nematodes co-cultured with hemolytic *B. cereus/thuringiensis* isolated from *P. wolfi* (Fig. 3b), but this impact was not seen when using hemolytic isolates from *P. moesta* (Fig. 3a).

### Growth inhibition of entomopathogenic fungi by *Bacillus* isolates

**Figure 4:**
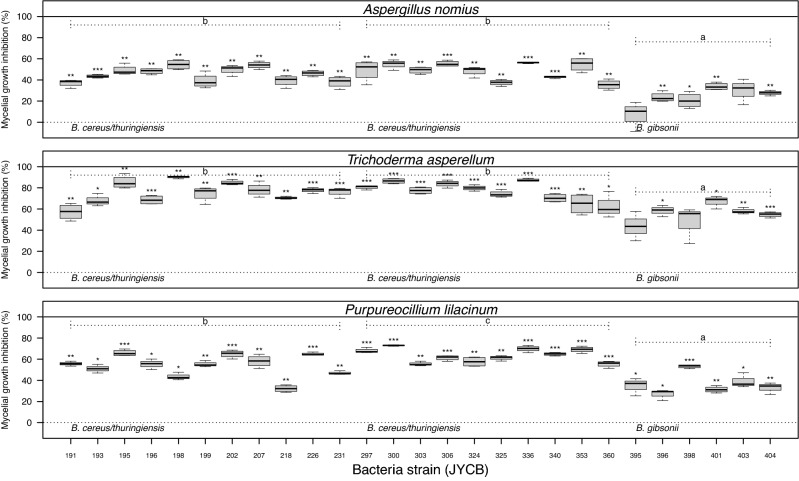
Mycelial growth inhibition (MGI) value of three entomopathogenic fungi under the effects of three predominant *Bacillus* strains isolated from ant cadavers infected by *Ophiocordyceps unilateralis *sensu lato. Letters indicate significant pairwise differences among the *Bacillus* species (Tukey’s all-pair comparisons using the beta regression model, *p* < 0.05). Starts indicate adjusted significant differences in comparing with 0.

Most of the bacterial strains examined in this study inhibited growth of the co-cultured entomopathogenic fungi significantly, although it was not statistically significant in a few of the strains, including JYCB395 and JYCB403 co-cultured with *A. nomius*, and JYCB395 and JYCB398 with *T. asperellum*. The strains that did not affect the fungal growth significantly belonged to clade w30, identified as *B. gibsonii*. The intensity of the growth inhibition of the three entomopathogenic fungi was different among the three bacterial species. (*A. nomius*: *X*^*2*^ = 87.21, *d.f.* = 2, *P* < 0.001; *T. asperellum*: *X*^*2*^ = 51.06, *d.f.* = 2, *P* < 0.001; *P. lilacinum*: *X*^*2*^ = 76.33, *d.f.* = 2, *P* < 0.001). The *B. cereus/thuringiensis* from each of the ant hosts inhibited growth of the entomopathogenic fungi noticeably, whereas *B. gibsonii* inhibited growth to a significantly lesser degree (Fig. 4).

### Resistance of *B. cereus/thuringiensis* to naphthoquinones

In a pretest with three randomly selected bacterial strains, the differences in the naphthoquinone tolerance in the bacteria isolated from the ants and the environment were more obvious with concentrations of 45 µg plumbagin/mL and 64.5 µg lapachol/mL (Fig. S3). These two concentrations were used in the following experiment.

**Figure 5:**
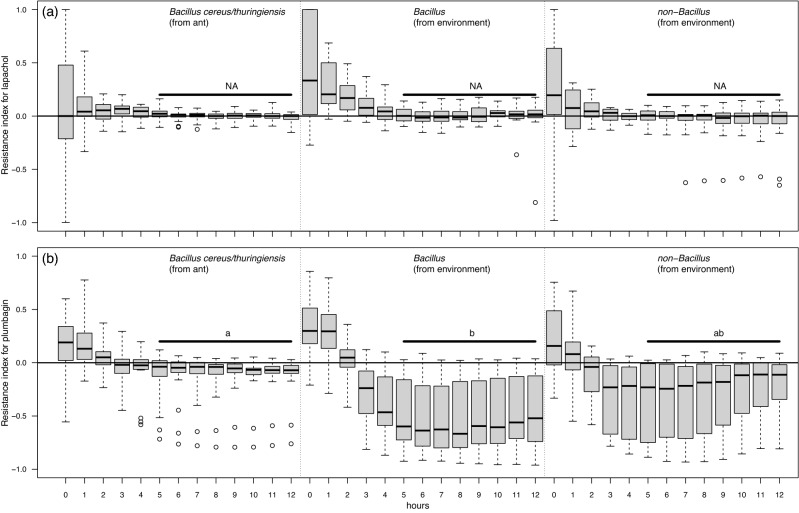
The resistance index of bacterial growth in the presence of two naphthoquinones, lapachol (**a**) and plumbagin (**b**). Letters indicate significant pairwise differences between *Bacillus cereus*/*thuringiensis* isolated from ant cadavers of *Polyrhachis moesta*, *Bacillus* isolates from the environment, and non-*Bacillus* isolates from the environment (Tukey’s all-pair comparisons using the linear mixed model, *p* < 0.05).

Bacterial isolates of all three categories (*B. cereus/thuringiensis* from the ant hosts, *Bacillus* from the environment, and non-*Bacillus* from the environment) displayed similar resistance to lapachol (*X*^*2*^ = 1.87, *d.f.* = 2, *P* = 0.392, Fig. 5a), growing similarly to bacteria cultured in the control medium (resistance indexes close to 1). In contrast, bacterial isolates from the environment, particularly the *Bacillus,* grew much slower in the presence of plumbagin than in the control medium, whereas *B. cereus/thuringiensis* from the ant host displayed a higher resistance to plumbagin (*X*^*2*^ = 6.91, *d.f.* = 2, *P* = 0.0316, Fig. 5b).

## Discussion

We found that *B. cereus/thuringiensis,* which occupied nearly 40% of the total bacterial counts, predominated the bacterial community in *O*. *unilateralis s*. *l*.-infected ant cadavers. In a study of the closely related fungus, *O. sinensi*s, the *Ophiocordyceps*-associated microbiomes improved the development and formation of fungal metabolites^13,14^. However, the main bacterial taxa found in the *O. sinensi*s-infected host cadavers was not *Bacillus*^13,14^. In the present study, the proliferation of *Bacillus* in ant cadavers could be from an invasion of the naphthoquinone-resistant population from soil. The genus *Bacillus* is one of the main bacterial groups in soil^43^. Furthermore, the predominant *B. cereus/thuringiensis* from ant cadavers displayed higher resistance to a specific naphthoquinone (plumbagin) than the bacteria isolated from the surrounding environment. Although such differences in the naphthoquinone effect have not been seen in lapachol, unequal antimicrobial activity has been found in previous studies [e.g., de Andrade-Neto et al. (2004)^44^]. In addition, the *B. cereus/thuringiensis* isolates from ant cadavers displayed hydrolytic enzyme activity, which suggests the potential to exploit resources of ant cadavers. Not surprisingly, lower bacterial diversity resulted in a higher abundance of *B. cereus/thuringiensis* in cadavers of the principal host, *P. moesta,* in comparison with the alternate sympatric host, *P. wolfi*. The accumulation of naphthoquinones might open a niche for *B. cereus/thuringiensis* isolates to outcompete other saprophytes, but the lower bacterial diversity in the principal host suggests a more sophisticated manipulation of the physiological conditions of the principal host. Despite not diverging among its sympatric hosts, *O. unilateralis s. l.* displays higher infection performances in one ant species than in the other host species in Taiwan^15^. The efficient exploitation of host resources may result from better modification of host physiological conditions, which consequently causes a more uniform environment for specific bacterial species. Fungi are known to modify the environment for their own benefit^2^. Some of the biological properties of the *B. cereus/thuringiensis* isolates, including in vitro hemolysis and antagonistic effects on co-cultured entomopathogenic fungi and nematodes, suggest the potential of a synergistic relationship with *O. unilateralis s. l.* However, because artificial infection of the ant host with *O*. *unilateralis s*. *l*. is difficult under our current laboratory conditions, to date we can only hypothesize about the potential synergistic effects of *B. cereus/thuringiensis* on *O*. *unilateralis s*. *l*. infections.

The *B. cereus/thuringiensis* isolates from both principal and alternate sympatric hosts are broadly aggressive to potential saprophytic invaders, including entomopathogenic fungi and nematodes. One possible factor is their hemolytic ability^45,46^. In comparison with non-hemolytic *Bacillus*, hemolytic *B. cereus/thuringiensis* isolates displayed higher fatal effects to free-living nematodes and growth inhibition of co-cultured entomopathogenic fungi. Collaborative bacteria have been known to assist parasites in occupying the host body by excluding potential invaders, such as nematode scavengers or insect predators^37,47,48^. For these collaborative bacteria, some of the antibiotic activity against invaders may be moderated by the need to compete with invaders for the necessary nutrition^49^, while not obviously affecting the host’s survival^50^. In contrast, for the *B. cereus/thuringiensis* isolates, in which host survival is no more a concern, the antibiotic activity could be more intensive and efficient. The *B. cereus/thuringiensis* isolates might play a role resembling that of the symbiotic bacteria, *Xenorhabdus* and *Photorhabdus*, in entomopathogenic nematodes. Invasion of the insect host by the nematode rapidly causes the insect’s death, and septicemia occurs with the proliferation of symbiotic bacteria. The nematodes then colonize the insect cadaver with the symbiotic bacteria, which now serve as “body guards” to defend against invasion by saprophytic or parasitic organisms^48^. Like the *B. cereus/thuringiensis* isolates, hemolysis is also detected in *X. nematophila* and likely plays a critical role in killing and preserving the insect host^51^. The outbreak of hemolytic *B. cereus/thuringiensis* isolates can be lethal to the ant host, and might explain the lack of endotoxin-related and biosynthetic genes in *O. unilateralis s. l.* due to overlapping functions. Another explanation for the lack of endotoxin-related genes is that the proliferation of *B. cereus/thuringiensis* occurs after the host death. However, the precise timing of the outbreak is currently unknown.

Regardless of the timing of *B. cereus/thuringiensis* proliferation, *B. cereus/thuringiensis* is potentially beneficial to *O*. *unilateralis s*. *l*. in protecting and consuming the host cadaver. *Bacillus* species produce several extracellular enzymes^52,53^, including alkaline proteases, which have been used commercially^54^. The ant cadaver contains nutritious and protein-rich niches for microbiota, but these are only made available in the presence of proteolytic enzymes, which digest the macromolecular proteins into smaller peptides and free amino acids^55^. The protease activity detected in all of the examined *B. cereus/thuringiensis* isolates suggests that symbiotic bacteria are advantageous to *O. unilateralis s. l.* in digesting the host tissue. In addition to improving consumption of the host, proteases are important for host epidermal decomposition and enhanced virulence to assist *O. unilateralis s. l.* in causing host death^56,57^. Chitin is another major component of insects that functions as a scaffolding material^58^. In addition to releasing nutrients, digesting chitin can be also fatal to the insect host and its invaders. Chitin is a necessary component of the peritrophic matrix secreted by the entire midgut in most insects, and it functions as a protective barrier against abrasive particles and microbial infections^59^. Chitinase secreted by bacteria weakens the insect’s peritrophic membrane, and consequently promotes the penetration of bacterial toxins to the gut epithelia during pathogenesis^60,61^. Chitinase produced by bacteria has also been found to present antagonistic activity against fungi, given that chitin is a primary component of fungal cell walls^62^. Digestion of lipids, in contrast to proteases and chitinase, might be less harmful for the living ant because lipids function mainly as storage structures. Nevertheless, lipids are a concentrated source of energy and primary nutrient reserves for fungal spores^63^. Extracellular lipid digestion by *B. cereus/thuringiensis* suggests that this bacterium efficiently harvests the energy from the ant cadaver and can symbiotically benefit *O. unilateralis s. l*. The hydrolytic enzymes, secreted to the microenvironment, may enhance the utilization of host resources. The importance of *B. cereus/thuringiensis* in consuming the host might be further supported by phylogenetic analysis of enzyme sequences. Unlike the genes for producing secondary metabolites, genes for producing hydrolytic enzymes in *O. unilateralis s. l.* display lower species specificity, which suggests a lack of positive selection among host species^64^. Sharing of the task by sympatric microorganisms reduces the indispensability and selective pressure on fungal hydrolytic enzymes.

The biological properties associated with an outbreak of *B. cereus/thuringiensis* suggest potential benefits to *O. unilateralis s. l*. The sympatric bacteria have recently been noted to play crucial roles in the growth of parasitoid fungi^65–67^. However, the proliferation of *B. cereus/thuringiensis* appears coincidental rather than the result of long-term coevolution. The bacterial communities in non-infected ants were not examined in this study. Despite the lack of data, *Bacillus* occupied a very low proportion of the bacterial community in living *Polyrhachis* ants based on the work of Ramalho et al. (2017)^68^ and Gatpatan et al. (2021)^69^. The predominance of *B. cereus/thuringiensis* may have originated from a small population in the living ant, the invasion of the bacteria from soil, or that carried by the fungal spore. Currently we do not have data supporting these hypotheses, but the outbreak of *B. cereus/thuringiensis* is still significant ecologically because it was detected in the two infected host species. In addition, the net effect of the inevitable competition with *B. cereus/thuringiensis* for limited resources may prove to be antagonistic rather than synergistic. Co-occurrence of fungi and bacteria can promote the growth of both entities^70^, but it can also accelerate the depletion of resources. Tradeoffs have been reported in fungi between growth and tolerance toward bacteria^1^. In this study, we demonstrated the predominance of *B. cereus/thuringiensis* in the bacterial community associated with ant cadavers infected by *O. unilateralis s. l*. These bacterial isolates displayed the capabilities of hemolysis, production of hydrolytic enzymes, antagonistic effects to co-cultured nematodes and entomopathogenic fungi, and higher tolerance toward naphthoquinone. At present we still do not have evidence to conclude that the outbreak of *B. cereus/thuringiensis* is antagonistic or synergistic toward *O. unilateralis s. l.* However, study of sympatric bacteria will improve our understanding of the parasitic life history and potential selective pressures in *O. unilateralis s. l*. In addition, the antibiotic activity of *B. cereus/thuringiensis* isolates has potential as a biocontrol agent. With antagonistic effects on entomopathogenic fungi and nematodes, *B. cereus/thuringiensis* also has potential agricultural applications in controlling pathogenic fungi^71^ and root-knot nematodes^72^. Through behavioral manipulation of the *O. unilateralis s. l.*–ant parasitic associations, the bacterial diversity revealed in this study is a step forward in understanding the impact of microbial communities in parasitic life cycles.

## Supplementary Information

Supplementary Information 1.

Supplementary Information 2.

Supplementary Information 3.

Supplementary Information 4.

Supplementary Information 5.

Supplementary Information 6.

Supplementary Information 7.

Supplementary Information 8.
