# Supplementary figures and images for: Possible impacts of the predominant Bacillus bacteria on the Ophiocordyceps unilateralis s. l. in its infected ant cadavers
Source: Sci Rep. 2021 Nov 22;11:22695. doi: 10.1038/s41598-021-02094-5 (PMC8609033; doi:10.1038/s41598-021-02094-5)

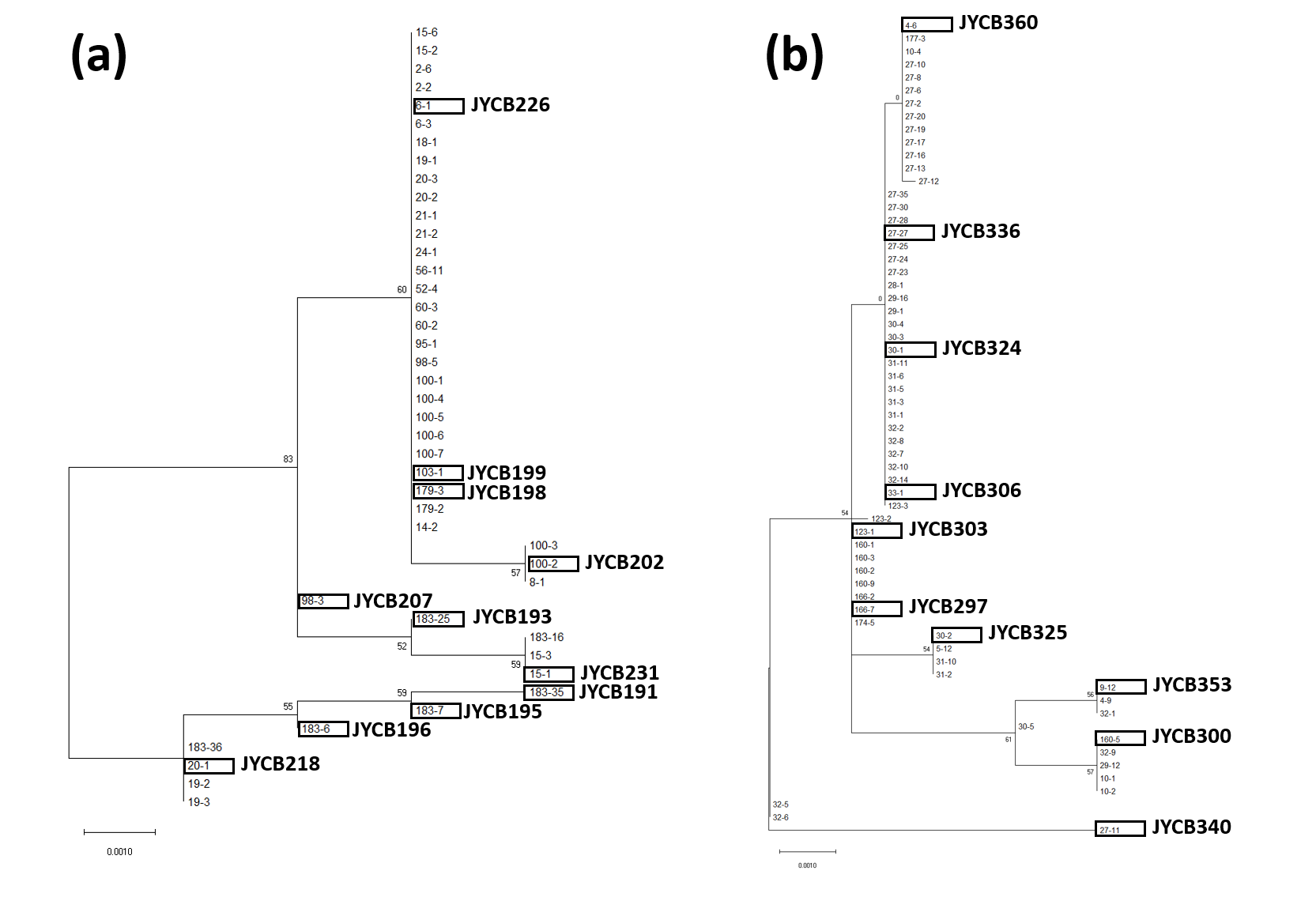

Supplement: Supplementary file 1 — Supplementary Information 1. [file 41598_2021_2094_MOESM1_ESM.tif]

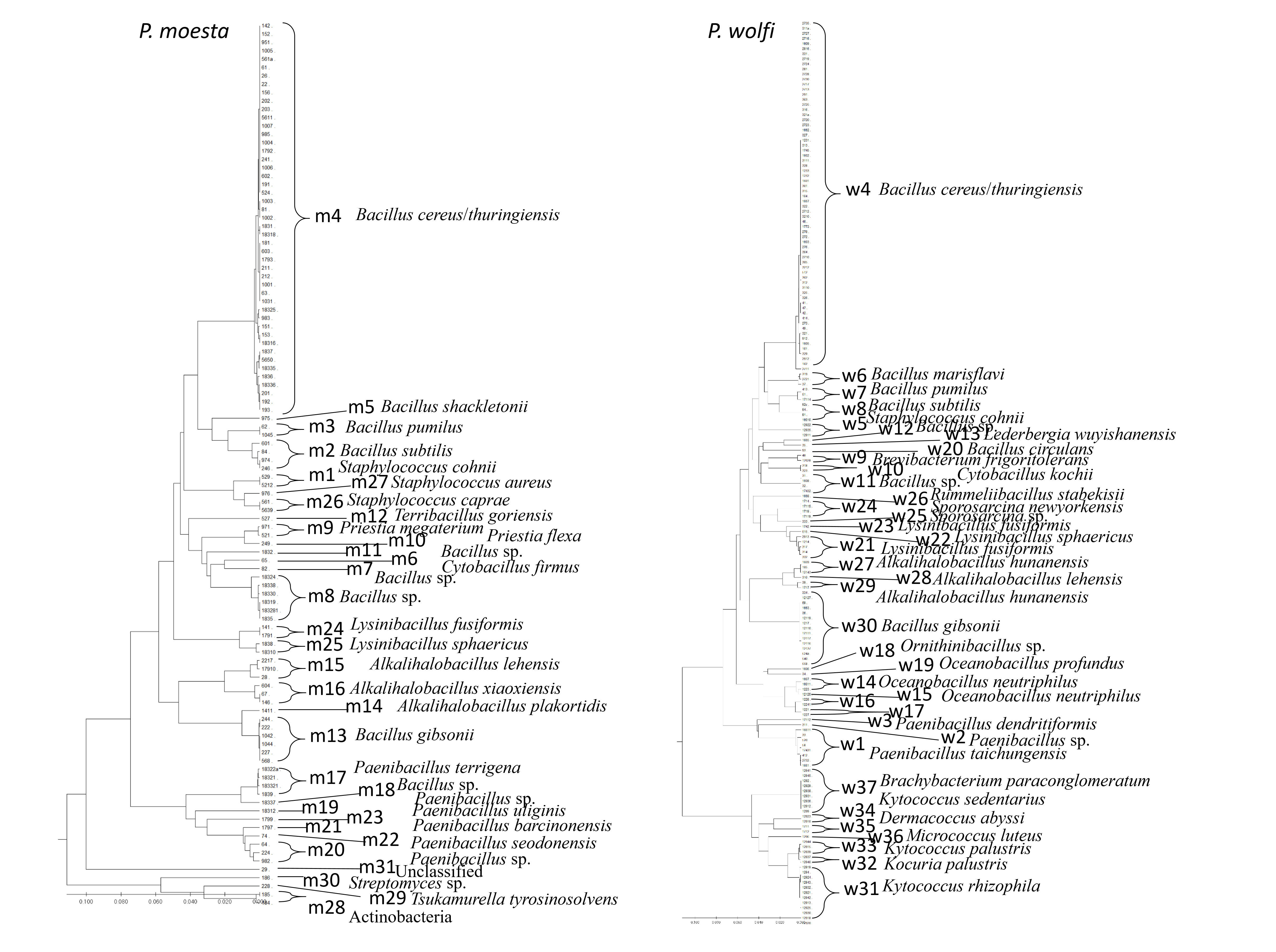

Supplement: Supplementary file 2 — Supplementary Information 2. [file 41598_2021_2094_MOESM2_ESM.tif]

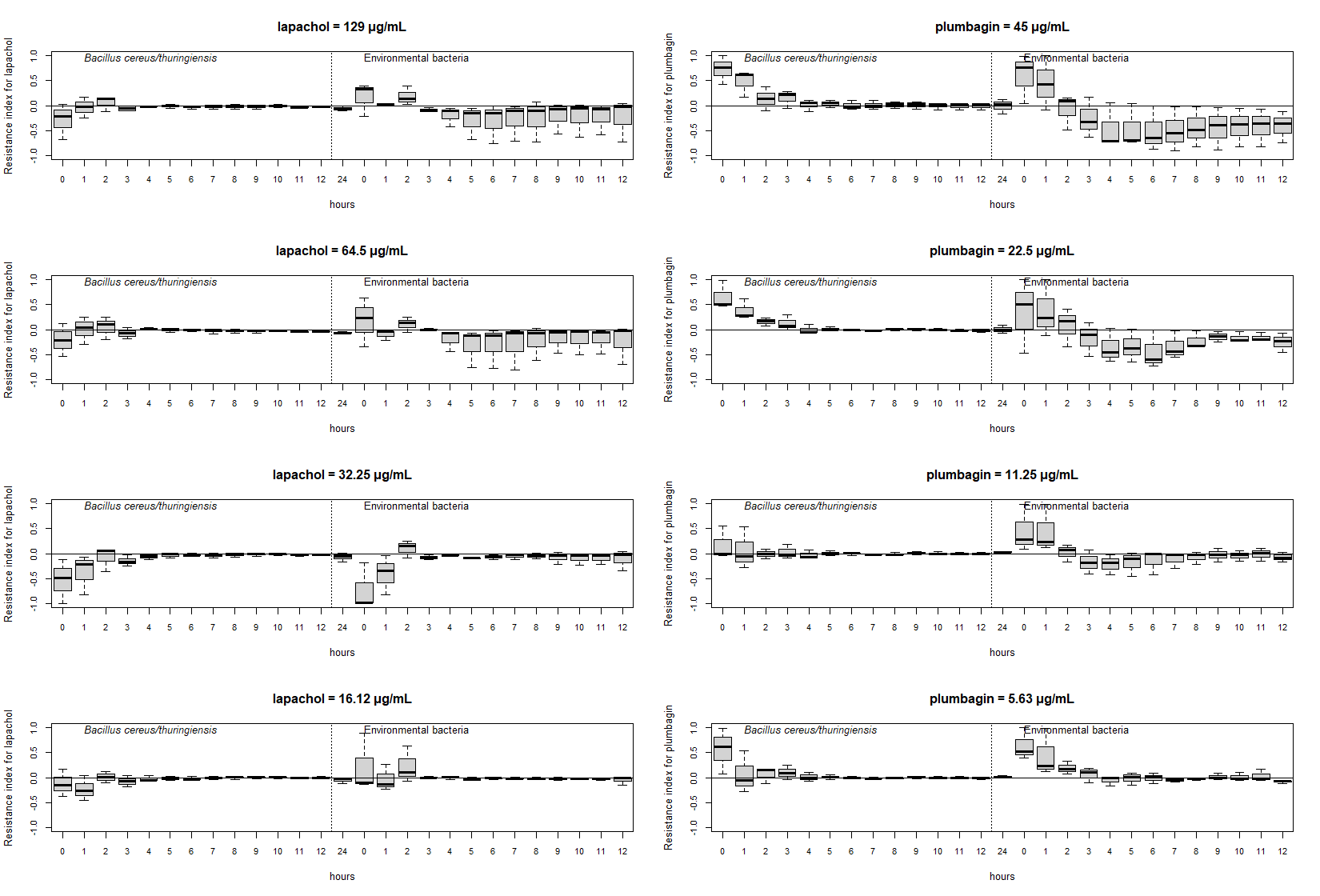

Supplement: Supplementary file 3 — Supplementary Information 3. [file 41598_2021_2094_MOESM3_ESM.tif]

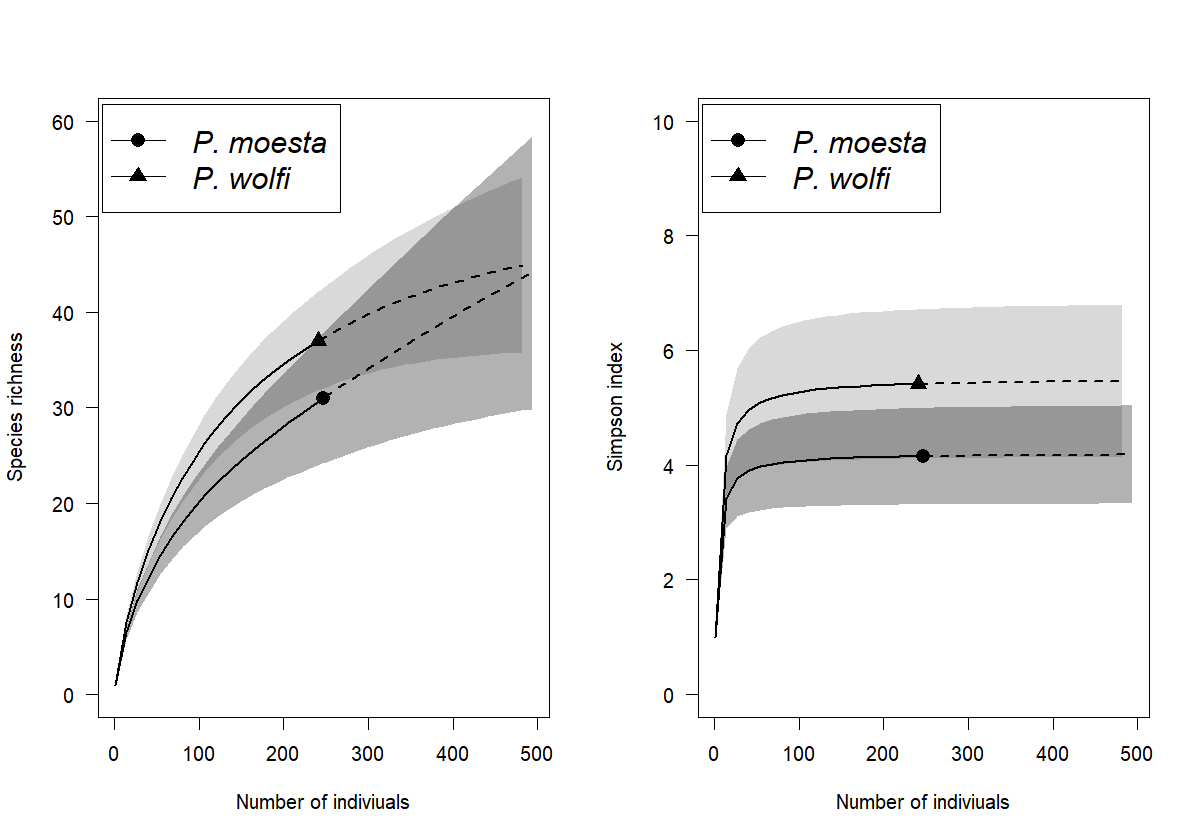

Supplement: Supplementary file 4 — Supplementary Information 4. [file 41598_2021_2094_MOESM4_ESM.tif]
