# Supplementary material for: Possible impacts of the predominant Bacillus bacteria on the Ophiocordyceps unilateralis s. l. in its infected ant cadavers
Source: Sci Rep. 2021 Nov 22;11:22695. doi: 10.1038/s41598-021-02094-5 (PMC8609033; doi:10.1038/s41598-021-02094-5)
Supplement: Supplementary file 5 — Supplementary Information 5. [file 41598_2021_2094_MOESM5_ESM.docx]

**Supplementary figure legend**

**Supplementary file 1.** 16S rDNA gene sequences of bacteria strains isolated from the *Ophiocordyceps unilateralis sensu lato* infected *Polyrhachis moesta* with the BLAST identity of estimated taxa and hemolytic activity. The columns “Clade” and “Isolate” are referred to the codes in **Supplementary Figure 1.**

**Supplementary file 2.** 16S rDNA gene sequences of bacteria strains isolated from the *Ophiocordyceps unilateralis sensu lato* infected *Polyrhachis wolfi* with the BLAST identity of estimated taxa and hemolytic activity. The columns “Clade” and “Isolate” are referred to the codes in **Supplementary Figure 1.**

**Supplementary Figure 1.** Strains of *Bacillus cereus*/*thuringiensis* from the ant cadavers of *Polyrhachis moesta* **(a)** and *P.* *wolfi* **(b)** selected for examining biological properties according to the UPGMA analysis by using MEGA X (https://www.megasoftware.net/).

**Supplementary Figure 2.** Clades of bacterial trains and their estimated taxa from the ant cadavers of *Polyrhachis moesta* **(a)** and *P.* *wolfi* **(b)**. Each of the clades are determined by the sequence dissimilarity (<0.01) according to the UPGMA analysis by using MEGA X (https://www.megasoftware.net/) and labeled the species names according to the BLAST result.

**Supplementary Figure 3.** Pre-test of naphthoquinone resistance to determine the naphthoquinone concentrations used in the experiment. Resistance index was calculated from six randomly selected bacteria (three *Bacillus cereus*/*thuringiensis* isolates from the ant hosts and three bacterial isolates from the environment) grown in the presence of two naphthoquinones (lapachol and plumbagin), with a serial dilution in 30% dimethyl sulfoxide (DMSO).

**Supplementary Figure 4.** Additional species diversity indexes of bacteria isolated from ant cadavers infected by *Ophiocordyceps unilateralis sensu lato*.
