# Supplementary material for: Possible impacts of the predominant Bacillus bacteria on the Ophiocordyceps unilateralis s. l. in its infected ant cadavers
Source: Sci Rep. 2021 Nov 22;11:22695. doi: 10.1038/s41598-021-02094-5 (PMC8609033; doi:10.1038/s41598-021-02094-5)
Supplement: Supplementary file 8 — Supplementary Information 8. [file 41598_2021_2094_MOESM8_ESM.docx]

Table S1. Bacterial clades (referring to the clades determined in Supplementary Figure 1.) from two infected ant hosts. **Genus** and **Species** are judged by the BLAST identity with the NCBI database. **Clade** (referring to the codes in Supplementary Figure 1.) are determined by the sequence dissimilarity (<0.01) of the bacterial strains according to the UPGMA analysis. **Abundance** means the sum of the colonies for the bacterial strains belonging to the same clade. **Hemolytic** is marked by +, -, and +/- which represent the hemolytic activity displayed in all, none, or some of the bacterial strains belonging to the same clade.

| **Bacteria isolated from infected *Polyrhachis moesta*** | | | | | **Bacteria isolated from infected *Polyrhachis wolfi*** | | | | | |
| --- | --- | --- | --- | --- | --- | --- | --- | --- | --- | --- |
| **Genus** | **Species** | **Clade** | **Abundance** | **Hemolytic** | **Genus** | **Species** | **Clade** | **Abundance** | | **Hemolytic** |
| **Phylum Firmicutes** | | | | | **Phylum Firmicutes** | | | | | |
| *Alkalihalobacillus* | *A. lehensis* | m15 | 3 | - | *Alkalihalobacillus* | *A. hunanensis* | w27 | 3 | | - |
|  | *A. plakortidis* | m14 | 1 | - |  | *A. hunanensis* | w29 | 2 | | **+/-** |
|  | *A. xiaoxiensis* | m16 | 4 | - |  | *A. lehensis* | w28 | 1 | | - |
| *Bacillus* | *B.* *cereus/thuringiensis* | m4 | 111 | **+/-** | *Bacillus* | *B. cereus/thuringiensis* | w4 | 94 | | **+/-** |
|  | *B. gibsonii* | m13 | 7 | - |  | *B. circulans* | w20 | 5 | | - |
|  | *B. pumilus* | m3 | 2 | **+/-** |  | *B. gibsonii* | w30 | 34 | | - |
|  | *B. shackletonii* | m5 | 1 | - |  | *B. marisflavi* | w6 | 3 | | **+/-** |
|  | *B. subtilis* | m2 | 4 | **+/-** |  | *B. pumilus* | w7 | 3 | | **+/-** |
|  | *Bacillus* sp. | m7 | 1 | - |  | *B. subtilis* | w8 | 15 | | **+** |
|  | *Bacillus* sp. | m8 | 8 | - |  | *Bacillus* sp. | w11 | 4 | | - |
|  | *Bacillus* sp. | m11 | 5 | - |  | *Bacillus* sp. | w12 | 1 | | - |
|  | *Bacillus* sp. | m18 | 1 | - | *Brevibacterium* | *B. frigoritolerans* | w9 | 5 | | - |
| *Cytobacillus* | *C. firmus* | m6 | 1 | - | *Cytobacillus* | *C. kochii* | w10 | 2 | | - |
| *Lysinibacillus* | *L. fusiformis* | m24 | 2 | + | *Lederbergia* | *L. wuyishanensis* | w13 | 1 | | - |
|  | *L. sphaericus* | m25 | 4 | **+/-** | *Lysinibacillus* | *L. fusiformis* | w21 | 5 | | **+/-** |
| *Paenibacillus* | *P. barcinonensis* | m21 | 1 | - |  | *L. fusiformis* | w23 | 1 | | - |
|  | *P. terrigena* | m17 | 4 | **+/-** |  | *L. sphaericus* | w22 | 1 | | - |
|  | *P. seodonensis* | m22 | 1 | - | *Oceanobacillus* | *O. neutriphilus* | w14 | 3 | | - |
|  | *P. uliginis* | m23 | 1 | - |  | *O. neutriphilus* | w15 | 1 | | - |
|  | *Paenibacillus* sp. | m19 | 1 | - |  | *O. profundus* | w19 | 1 | | - |
|  | *Paenibacillus* sp. | m20 | 3 | - | *Ornithinibacillus* | *Ornithinibacillus* sp. | w18 | 1 | | - |
| *Priestia* | *P. flexa* | m10 | 1 | - | *Paenibacillus* | *P. dendritiformis* | w3 | 1 | | **+** |
|  | *P. megaterium* | m9 | 3 | - |  | *P. taichungensis* | w1 | 9 | | - |
| *Staphylococcus* | *S. aureus* | m27 | 1 | **+** |  | *Paenibacillus* sp. | w2 | 1 | | - |
|  | *S. caprae* | m26 | 64^1^ | - | *Rummeliibacillus* | *R. stabekisii* | w26 | 1 | | - |
|  | *S. cohnii* | m1 |  | - | *Sporosarcina* | *S. newyorkensis* | w24 | 4 | | - |
| *Terribacillus* | *T. goriensis* | m12 | 1 | - |  | *Sporosarcina* sp. | w25 | 1 | | - |
| **Phylum Actinobacteria** | | | | | *Staphylococcus* | *S. cohnii* | w5 | 3 | - | |
| *Streptomyces* | *Streptomyces* sp. | m30 | 1 | - | Unclassified |  | w16 | 2 | | - |
| *Tsukamurella* | *T. tyrosinosolvens* | m29 | 1 | - |  |  | w17 | 2 | |  |
| Unclassified |  | m28 | 8 | - | **Phylum Actinobacteria** | | | | | |
| **Unclassified bacterium** | | | | | *Brachybacterium* | *B. paraconglomeratum* | w37 | 9 | - | |
| Unclassified |  | m31 | 1 | - | *Dermacoccus* | *D. abyssi* | w35 | 2 | | - |
|  |  |  |  |  | *Kocuria* | *K. palustris* | w32 | 2 | | - |
|  |  |  |  |  | *Kytococcus* | *K. sedentarius* | w34 | 2 | | - |
|  |  |  |  |  |  | *K. palustris* | w33 | 3 | | - |
|  |  |  |  |  |  | *K. rhizophila* | w31 | 12 | | - |
|  |  |  |  |  | *Micrococcus* | *M. luteus* | w36 | 1 | | - |

^1^Raw data of the clades, m26 and m1, were loosed and only the summarized abundance of the two clades is currently known. In the calculation of the biodiversity, each of the clades was considered as 32 individuals in the abundance which makes the possible over-estimation of the biodiversity in *Polyrhachis moesta*. Despite it, the over-estimated biodiversity is still lower than that in *Polyrhachis wolfi* which did not influence the conclusion in this study.

Table S2 Primers for pathogenic and antibiotic genes

| **Genes** | **Primer** | | | **Reference** |
| --- | --- | --- | --- | --- |
|  | **name** | **F** | **R** |  |
| *cry1* | Un1 | CATGATTCATGCGGCAGATAAAC | TTGTGACACTTCTGCTTCCCATT | (Jain et al., 2017) |
| *cry2* | Un2 | GTTATTCTTAATGCAGATGAATGGG | CGGATAAAATAATCTGGGAAATAGT | (Jain et al., 2017) |
| *cry3* | Un3 | CGTTATCGCAGAGAGATGACATTAAC | CATCTGTTGTTTCTGGAGGCAAT | (Jain et al., 2017) |
| *cry4* | Un4 | GCATATGATGTAGCGAAACAAGCC | GCGTGACATACCCATTTCCAGGTCC | (Jain et al., 2017) |
| *cry5, 12, 14, 21* | Un5 | TTACGTAAATTGGTCAATCAAGCAAA | AAGACCAAATTCAATACCAGGGTT | (Jain et al., 2017) |
| *cry7, 8* | Un7-8 | AAGCAGTGAATGCCTTGTTTAC | CTTCTAAACCTTGACTACTT | (Jain et al., 2017) |
| *cry9* | Un9 | CGGTGTTACTATTAGCGAGGGCGG | GTTTGAGCCGCTTCACAGCAATCC | (Jain et al., 2017) |
| *cry11* | Un11 | TTCCAACCCAACTTTCAAGC | AGCTATGGCCTAAGGGGAAA | (Jain et al., 2017) |
| *vip3* | Vip | CCTCTATGTTGAGTGATGTA | CTATACTCCGCTTCACTTGA | (Jain et al., 2017) |
| *cyt1* | Cyt1A | AACCCCTCAATCAACAGCAAGG | GGTACACAATACATAACGCCACC | (Jain et al., 2017) |
| *cyt1* | Cyt1B | CCTCAATCAACAGCAAGGGTTATT | TGCAAACAGGACATTGTATGTGTAATT | (Costa et al., 2014) |
| *cyt2* | Cyt2A | AATACATTTCAAGGAGCTA | TTTCATTTTAACTTCATATC | (Jain et al., 2017) |
| *cyt2* | Cyt2B | ATTACAAATTGCAAATGGTATTCC | TTTCAACATCCACAGTAATTTCAAATGC | (Costa et al., 2014) |
| Iturin | ItuD1 | GATGCGATCTCCTTGGATGT | ATCGTCATGTGCTGCTTGAG | (Athukorala et al., 2009) |
| Iturin | ituD | TTGAAYGTCAGYGCSCCTTT | TGCGMAAATAATGGSGTCGT | (Płaza et al., 2015) |
| Iturin | ituC | GGCTGCTGCAGATGCTTTAT | TCGCAGATAATCGCAGTGAG | (Płaza et al., 2015) |
| Iturin | ituC-1 | CCCCCTCGGTCAAGTGAATA | TTGGTTAAGCCCTGATGCTC | (Chung et al., 2008) |
| chitinase | chi | ATGGTCATGAGGTCTC | CTATTTCGCTAATGACG | (Soares-da-Silva et al., 2017) |
| Bacillomycin | Bacc1 | GAAGGACACGGAGAGAGTC | CGCTGATGACTGTTCATGCT | (Athukorala et al., 2009) |
| Fengycin | FenD1 | TTTGGCAGCAGGAGAAGTTT | GCTGTCCGTTCTGCTTTTTC | (Athukorala et al., 2009) |
| Fengycin | fenB | CCTGGAGAAAGAATATACCGTACCY | GCTGGTTCAGTTKGATCACAT | (Płaza et al., 2015) |
| Fengycin | fenD | GGCCCGTTCTCTAAATCCAT | GTCATGCTGACGAGAGCAAA | (Płaza et al., 2015) |
| Surfactin | Sur3 | ACAGTATGGAGGCATGGTC | TTCCGCCACTTTTTCAGTTT | (Athukorala et al., 2009) |
| Surfactin | SrfAA | TCGGGACAGGAAGACATCAT | CCACTCAAACGGATAATCCTGA | (Płaza et al., 2015) |
| Surfactin | Sfp | ATGAAGATTTACGGAATTTA | TTATAAAAGCTCTTCGTACG | (Płaza et al., 2015) |
| Surfactin | SrfA-1 | AGAGCACATTGAGCGTTACAAA | CAGCATCTCGTTCAACTTTCAC | (Chung et al., 2008) |
| Zwittermicin A | ZWIT | TTGGGAGAATATACAGCTCT | GACCTTTTGAAATGGGCGTA | (Athukorala et al., 2009) |

1. Athukorala, S. N., Fernando, W. D. & Rashid, K. Y. Identification of antifungal antibiotics of *Bacillus* species isolated from different microhabitats using polymerase chain reaction and MALDI-TOF mass spectrometry. *Can. J. Microbiol.* **55**, 1021–1032. <https://doi.org/10.1139/w09-067> (2009).
2. Chung, S., Kong, H., Buyer, J. S., Lakshman, D. K., Lydon, J., Kim, S. D., & Roberts, D. P. Isolation and partial characterization of *Bacillus* *subtilis* ME488 for suppression of soilborne pathogens of cucumber and pepper. *Appl. Microbiol. Biotechnol.* **80**, 115–223. <https://doi.org/10.1007/s00253-008-1520-4> (2008).
3. Costa, M. L. M., Lana, U. G. P., Barros, E. C., Paiva, L. V. & Valicente, F. H. Molecular characterization of *Bacillus* *thuringiensis* cyt genes and their effect against fall armyworm, *Spodoptera frugiperda*. *J. Agric. Sci.* **6**, 128–137. <https://doi.org/10.5539/jas.v6n7p128> (2014).
4. Jain, D., Sunda, S. D., Sanadhya, S., Nath, D. J. & Khandelwal, S. K. Molecular characterization and PCR-based screening of cry genes from *Bacillus thuringiensis* strains. *3 Biotech.* **7**, 4. <https://doi.org/10.1007/s13205-016-0583-7> (2017).
5. Płaza, G., Chojniak, J., Rudnicka, K., Paraszkiewicz, K., & Bernat, P. Detection of biosurfactants in *Bacillus* species: genes and products identification. *J. Appl. Microbiol.* **119**, 1023–1034. <https://doi.org/10.1111/jam.12893> (2015).
6. Soares-da-Silva, J., Queirós, S. G., de Aguiar, J. S., Viana, J. L., Neta, M. D. R. A. V, da Silva, M. C., Pinheiro, V. C. S, Polanczyk, R. A., Carvalho-Zilse, G. A., & Tadei, W. P. Molecular characterization of the gene profile of *Bacillus thuringiensis* Berliner isolated from Brazilian ecosystems and showing pathogenic activity against mosquito larvae of medical importance. *Acta. Trop.* **176**, 197–205. <https://doi.org/10.1016/j.actatropica.2017.08.006> (2017).

Table S3 Pathogenic and antibiotic genes (*cry*, *vip*, *cyt*, Iturin A)

| **Bacteria isolats** | ***cry*** | | | | | | | | ***vip*** | ***cyt*** | | | | **Iturin** | | |
| --- | --- | --- | --- | --- | --- | --- | --- | --- | --- | --- | --- | --- | --- | --- | --- | --- |
|  | **Un1** | **Un2** | **Un3** | **Un4** | **Un5** | **Un7-8** | **Un9** | **Un11** | **Vip** | **Cyt1A** | **Cyt2A** | **Cyt1B** | **Cyt2B** | **ItuD1** | **ItuD** | **ItuC** |
| *Bacillus thuringiensis* JYCB226 | - | - | - | - | - | - | - | - | - | - | - | - | - | - | - | - |
| *Bacillus thuringiensis* JYCB218 | - | - | - | - | - | - | - | - | - | - | - | - | - | - | - | - |
| *Bacillus thuringiensis* JYCB202 | - | - | - | - | - | - | - | - | - | - | - | - | - | - | - | - |
| *Bacillus thuringiensis* JYCB198 | - | - | - | - | - | - | - | - | - | - | - | - | - | - | - | - |
| *Bacillus thuringiensis* JYCB195 | - | - | + | - | - | - | - | - | - | - | - | - | - | - | - | - |
| *Bacillus thuringiensis* JYCB199 | - | - | + | - | - | - | - | - | - | - | - | - | - | - | - | - |
| *Bacillus thuringiensis* JYCB193 | - | - | - | - | - | - | - | - | - | - | - | - | - | - | - | - |
| *Bacillus thuringiensis* JYCB191 | - | - | - | - | - | - | - | - | - | - | - | - | - | - | - | - |
| *Bacillus thuringiensis* JYCB207 | - | - | - | - | - | - | - | - | - | - | - | - | - | - | - | - |
| *Bacillus thuringiensis* JYCB196 | - | - | - | - | - | - | - | - | - | - | - | - | - | - | - | - |
| *Bacillus thuringiensis* JYCB231 | - | - | - | - | - | - | - | - | - | - | - | - | - | - | - | - |
| *Bacillus thuringiensis* JYCB324 | - | - | - | - | - | - | - | - | - | - | - | - | - | - | - | - |
| *Bacillus thuringiensis* JYCB306 | - | - | - | - | - | - | - | - | - | - | - | - | - | - | - | - |
| *Bacillus thuringiensis* JYCB303 | - | - | - | - | - | - | - | - | - | - | - | - | - | - | - | - |
| *Bacillus thuringiensis* JYCB336 | - | - | - | - | - | - | - | - | - | - | - | - | - | - | - | - |
| *Bacillus thuringiensis* JYCB300 | - | - | - | - | - | - | - | - | - | - | - | - | - | - | - | - |
| *Bacillus thuringiensis* JYCB297 | - | - | - | - | - | - | - | - | - | - | - | - | - | - | - | - |
| *Bacillus thuringiensis* JYCB360 | - | - | - | - | - | - | - | - | - | - | - | - | - | - | - | - |
| *Bacillus thuringiensis* JYCB353 | - | - | - | - | - | - | - | - | - | - | - | - | - | - | - | - |
| *Bacillus thuringiensis* JYCB325 | - | - | - | - | - | - | - | - | - | - | - | - | - | - | - | - |
| *Bacillus thuringiensis* JYCB340 | - | - | - | - | - | - | - | - | - | - | - | - | - | - | - | - |
| *Bacillus gibsonii* JYCB395 | - | - | - | - | - | - | - | - | - | - | - | - | - | - | - | - |
| *Bacillus gibsonii* JYCB396 | - | - | - | - | - | - | - | - | - | - | - | - | - | - | - | - |
| *Bacillus gibsonii* JYCB398 | - | - | - | - | - | - | - | - | - | - | - | - | - | - | - | - |
| *Bacillus gibsonii* JYCB403 | - | - | - | - | - | - | - | - | - | - | - | - | - | - | - | - |
| *Bacillus gibsonii* JYCB404 | - | + | - | - | - | - | - | - | - | - | - | - | - | - | - | - |
| *Bacillus gibsonii* JYCB401 | - | - | - | - | - | - | - | - | - | - | - | - | - | - | - | - |

Table S4 Pathogenic and antibiotic genes (chitinase, Bacillomycin D, Fengycin, Surfactin, Zwittermicin A)

| **Bacteria isolats** | **chitinase** | **Bacillomycin D** | **Fengycin** | | | **Surfactin** | | | | **Zwittermicin A** |
| --- | --- | --- | --- | --- | --- | --- | --- | --- | --- | --- |
|  | **chi** | **Bacc1** | **FenD1** | **fenB** | **fenD** | **Sur3** | **SrfAA** | **Sfp** | **SrfA-1** | **ZWIT** |
| ***Bacillus* species isolated from infected *P*. *moesta*** | | | | | | | | | | |
| *Bacillus thuringiensis* JYCB226 | + | - | - | - | - | - | - | - | - | + |
| *Bacillus thuringiensis* JYCB218 | - | - | - | - | - | - | - | - | - | - |
| *Bacillus thuringiensis* JYCB202 | + | - | - | - | - | - | - | - | - | - |
| *Bacillus thuringiensis* JYCB198 | - | - | - | - | - | - | - | - | - | - |
| *Bacillus thuringiensis* JYCB195 | - | - | - | - | - | - | + | - | - | - |
| *Bacillus thuringiensis* JYCB199 | + | - | - | - | - | - | - | - | - | - |
| *Bacillus thuringiensis* JYCB193 | - | - | - | - | - | - | - | - | - | - |
| *Bacillus thuringiensis* JYCB191 | - | - | - | - | - | - | + | - | - | - |
| *Bacillus thuringiensis* JYCB207 | + | - | - | - | - | - | - | - | - | - |
| *Bacillus thuringiensis* JYCB196 | - | - | - | - | - | + | - | - | - | - |
| *Bacillus thuringiensis* JYCB231 | - | - | - | - | - | + | - | - | - | - |
| ***Bacillus* species isolated from infected *P*. *wolfi*** | | | | | | | | | | |
| *Bacillus thuringiensis* JYCB324 | - | - | - | - | - | - | - | - | - | - |
| *Bacillus thuringiensis* JYCB306 | - | - | - | - | - | - | - | - | - | - |
| *Bacillus thuringiensis* JYCB303 | - | - | - | - | - | - | - | - | - | - |
| *Bacillus thuringiensis* JYCB336 | - | - | - | - | - | - | - | - | - | - |
| *Bacillus thuringiensis* JYCB300 | + | - | - | - | - | - | - | - | - | - |
| *Bacillus thuringiensis* JYCB297 | - | - | - | - | - | - | - | - | - | - |
| *Bacillus thuringiensis* JYCB360 | - | - | - | - | - | - | - | - | - | - |
| *Bacillus thuringiensis* JYCB353 | - | - | - | - | - | - | - | - | - | - |
| *Bacillus thuringiensis* JYCB325 | - | - | - | - | - | - | - | - | - | - |
| *Bacillus thuringiensis* JYCB340 | + | - | - | - | - | - | - | - | - | - |
| *Bacillus gibsonii* JYCB395 | - | - | - | - | - | - | - | - | - | - |
| *Bacillus gibsonii* JYCB396 | - | - | - | - | - | - | - | - | - | - |
| *Bacillus gibsonii* JYCB398 | - | - | - | - | - | - | - | - | - | - |
| *Bacillus gibsonii* JYCB403 | - | - | - | - | - | - | - | - | - | - |
| *Bacillus gibsonii* JYCB404 | + | - | - | - | - | - | - | - | - | - |
| *Bacillus gibsonii* JYCB401 | - | - | - | - | - | - | - | - | - | - |

Table S5 Chitinase、protease、lipase and esterase activity of *Bacillus* isolates from infected *P*. *moesta* and *P*. *wolfi*.

| **Bacteria isolats** | ***chitinase* (28℃)** | ***protease*** | ***lipase*** | ***esterase*** |
| --- | --- | --- | --- | --- |
| ***Bacillus* species isolated from infected *P*. *moesta*** | | | | |
| *Bacillus thuringiensis* JYCB226 | No growth | + | + | + |
| *Bacillus thuringiensis* JYCB218 | No growth | + | + | + |
| *Bacillus thuringiensis* JYCB202 | No growth | + | + | + |
| *Bacillus thuringiensis* JYCB198 | No growth | + | + | + |
| *Bacillus thuringiensis* JYCB195 | No growth | + | + | + |
| *Bacillus thuringiensis* JYCB199 | No growth | + | + | + |
| *Bacillus thuringiensis* JYCB193 | No growth | + | + | + |
| *Bacillus thuringiensis* JYCB191 | No growth | + | + | + |
| *Bacillus thuringiensis* JYCB207 | No growth | + | + | + |
| *Bacillus thuringiensis* JYCB196 | No growth | + | + | + |
| *Bacillus thuringiensis* JYCB231 | No growth | + | + | + |
| ***Bacillus* species isolated from infected *P*. *wolfi*** | | | | |
| *Bacillus thuringiensis* JYCB324 | + | + | + | + |
| *Bacillus thuringiensis* JYCB306 | + | + | + | + |
| *Bacillus thuringiensis* JYCB303 | + | + | + | + |
| *Bacillus thuringiensis* JYCB336 | + | + | + | + |
| *Bacillus thuringiensis* JYCB300 | + | + | + | + |
| *Bacillus thuringiensis* JYCB297 | + | + | + | + |
| *Bacillus thuringiensis* JYCB360 | + | + | + | + |
| *Bacillus thuringiensis* JYCB353 | + | + | + | + |
| *Bacillus thuringiensis* JYCB325 | + | + | + | + |
| *Bacillus thuringiensis* JYCB340 | + | + | + | + |
| *Bacillus gibsonii* JYCB395 | No growth | No growth | + | No growth |
| *Bacillus gibsonii* JYCB396 | No growth | No growth | + | No growth |
| *Bacillus gibsonii* JYCB398 | No growth | No growth | + | No growth |
| *Bacillus gibsonii* JYCB403 | No growth | No growth | + | No growth |
| *Bacillus gibsonii* JYCB404 | No growth | No growth | + | No growth |
| *Bacillus gibsonii* JYCB401 | No growth | No growth | + | No growth |
